# Acceptability of a hypothetical dengue vaccine and the potential impact of dengue vaccination on personal vector control behavior: a qualitative study in Fortaleza, Brazil

**DOI:** 10.1186/s12889-023-17005-8

**Published:** 2023-12-04

**Authors:** Valerie K. Scott, Maria Suelly Nogueira Pinheiro, Marcia Maria Tavares Machado, Marcia C. Castro

**Affiliations:** 1 Department of Global Health and Population, Harvard T.H. Chan School of Public Health, 677 Huntington Ave, 02115 Boston, MA USA; 2 Federal University of Ceará (UFC), Fortaleza, Ceará Brazil

**Keywords:** Dengue vaccines, Vaccine acceptance, Vaccine hesitancy, Vector control

## Abstract

**Background:**

Dengue is the most rapidly spreading viral vector-borne disease in the world. Promising new dengue vaccines have contributed to a growing consensus that effective dengue control will require integrated strategies of vaccination and vector control. In this qualitative study, we explored the perspectives of residents of Fortaleza, Brazil on acceptability of a hypothetical safe and effective dengue vaccine, specific drivers of dengue vaccine acceptance or hesitance, and the expected impact of dengue vaccination on their personal vector control practices.

**Methods:**

A total of 43 in-depth interviews were conducted from April to June 2022 with Fortaleza residents from a diverse range of educational and professional backgrounds, with and without recent personal experiences of symptomatic dengue infections. Data were analyzed using the principles of inductive grounded theory methodology.

**Results:**

Our findings indicate that knowledge of dengue transmission, symptoms, and prevention methods was strong across respondents. Respondents described willingness to accept a hypothetical dengue vaccine for themselves and their children, while emphasizing that the vaccine must be demonstrably safe and effective. Respondents expressed diverse perspectives on how receiving a safe and effective dengue vaccine might influence their personal vector control behaviors, relating these behaviors to their perception of risk from other *Aedes* mosquito*-*carried infections and beliefs about the role of vector control in maintaining household cleanliness.

**Conclusions:**

Our study findings provide community-level perspectives on dengue vaccination and its potential impact on personal vector control behavior for policymakers and program managers in Fortaleza to consider as new dengue vaccines become available. With the introduction of any new dengue vaccine, community perspectives and emerging concerns that may drive vaccine hesitancy should be continuously sought out. Improved urban infrastructure and efforts to engage individuals and communities in vector control may be needed to optimize the impact of future dengue vaccinations and prevent rising cases of other arboviruses such as Zika and chikungunya.

## Background

Dengue is the most rapidly spreading viral vector-borne disease in the world. It is an arboviral disease transmitted between humans by *Aedes* mosquitoes [1]. Four genetically distinct serotypes of dengue virus (DENV 1–4) exist, often co-circulating in the same region [2]. More than 2.8 million dengue cases were reported in the Americas region in 2022, with most of these cases (2,383,001) occurring in Brazil. Dengue circulates together with chikungunya and Zika, arboviruses also carried by the *Aedes aegypti* mosquito, and the SARS-CoV-2 virus in the region [3].

Rapid urbanization, poor water and sanitation infrastructure, and global trade and travel networks have contributed to the proliferation of *Aedes* mosquitoes and worsening dengue outbreaks around the world [4, 5]. *Ae. aegypti* mosquitoes, the principal dengue vector, are highly adapted to human habitats, biting during the day and often breeding in human-made containers that hold water near and inside people’s homes. *Ae. albopictus* are a less efficient dengue vector, but their geographic expansion in tropical and temperate climates may introduce arboviral risk in new areas [6].

Most dengue infections are subclinical, with no or very mild symptoms; however, roughly 25% of infected people develop febrile illness [7, 8]. A small proportion of symptomatic dengue patients develop severe dengue—rarely, this can be fatal, and only supportive care is available currently [9, 10]. Becoming infected with a new serotype of dengue after an initial infection is a major risk factor for developing severe dengue [11].

Without an effective antiviral treatment or, until recently, an effective vaccine, dengue control has historically centered on vector control efforts, including environmental, chemical, and biological interventions. Outdoor spraying operations target adult mosquitoes, while environmental management strategies—for example, removing or covering water storage containers—aim to prevent dengue transmission by targeting larval stages of *Ae. aegypti.* Rigorous evidence on the effectiveness of vector control strategies in reducing dengue transmission is scarce [12]. Cluster randomized controlled trials in Brazil (Fortaleza), Cuba, and India have demonstrated that community-based environmental management strategies can effectively reduce *Ae. aegypti* populations [13–15]. Based on these studies, investing in community engagement and mobilization activities such as establishing community working groups to identify priorities and create action plans, implementing community clean-up days and encouraging individuals to cover water containers, and launching information and behavior change campaigns appears to have a meaningful impact on vector population densities. Vanlerberghe et al.’s community-based environmental management intervention in Cuba included a component of working with the local government to improve waste and sewage systems, highlighting how individual and community-based efforts can be combined with larger-scale improvements in infrastructure to effectively target the *Ae. aegypti* mosquito population [15].

Recent promising dengue vaccine developments have supported a growing consensus that effectively combatting dengue will require integrated strategies of vaccination and vector control. In 2015, Sanofi Pasteur’s Dengvaxia, the world’s first dengue vaccine, was licensed and is now registered in 20 countries. Dengvaxia reduced the incidence of symptomatic dengue infection and hospitalization [16, 17], but vaccine efficacy varied by virus serotype, patient baseline serostatus, and patient age [18]. Significant controversy followed an announcement by Sanofi in November 2017 that there was a heightened risk of severe dengue among individuals who were seronegative (not previously exposed to dengue) when vaccinated. This finding led the World Health Organization (WHO) to recommend pre-vaccination screening, with only seropositive individuals receiving Dengvaxia. In Brazil, Dengvaxia had been introduced in only one state (Paraná) as part of a pilot program aiming to vaccinate 500,000 individuals from 2016 to 2018 and following the WHO’s updated recommendation, its use was restricted to seropositive individuals [19].

In 2023, Brazil’s National Health Surveillance Agency (ANVISA) approved a new dengue vaccine—Takeda Pharmaceutical’s Qdenga (TAK-003)—for use in individuals aged 4 to 60 years, regardless of prior dengue exposure [20]. Phase 3 clinical trial results showed that Qdenga had a cumulative efficacy of 83.6% against hospitalization with virologically confirmed dengue and 62.0% against virologically confirmed dengue in an overall study population of both seropositive and seronegative individuals [21]. Another dengue vaccine, the Butantan-DV vaccine (analogous to the U.S. National Institutes of Health TV003 vaccine) is also currently in the final stages of a randomized controlled Phase 3 trial in Brazil [22].

Given this substantial progress in vaccine development, Brazil will likely introduce a new dengue vaccine in the near-term future. Novel dengue vaccines around the world will be introduced in a particularly challenging context marked by rising global vaccine hesitancy, the spread of misinformation and mistrust, and the increasing politicization of vaccines with the COVID-19 pandemic [23]. Brazil’s National Immunization Program (*Programa Nacional de Imunizações –* PNI) integrated into the National Health System (*Sistema Único de Saúde –* SUS) has been considered a global model for vaccine coverage for several decades [24, 25]. However, in recent years, the anti-vaccine movement in Brazil has been growing, and vaccine confidence and overall vaccination coverage of children has declined [25]. Few studies have been conducted to assess dengue vaccine acceptance [26, 27] and willingness-to-pay (as a measure of demand) for a dengue vaccine [26, 28–30].

Further, there is little research available on the potential impact of dengue vaccination on personal vector control behaviors [26]. Particularly in countries such as Brazil with co-circulating arboviruses carried by *Aedes* mosquitoes, the possible influence of dengue vaccination on community-level vector control will need to be considered, with measures taken to mitigate any increased risk of other arboviral transmission. New dengue vaccine programs will likely have gaps in protection and coverage that vary over space and time, and endemic communities with high levels of dengue vaccination may continue to be exposed to risk from other *Aedes-*carried arboviruses.

This qualitative study conducted in Fortaleza, Brazil aimed to better understand public acceptability of a hypothetical safe and effective dengue vaccine and identify specific drivers of vaccine acceptance or hesitance. We also sought to explore the potential influence of dengue vaccination on personal vector control practices. This study’s findings provide community-level perspectives on dengue vaccination and its interplay with personal vector control behavior for policymakers, program managers, and healthcare providers to consider as they design and implement dengue vaccination and vector control programs in Fortaleza.

## Methods

### Study design

We designed and analyzed this qualitative study using the principles of grounded theory—a qualitative research design in which theories, or explanations of processes, are generated or “grounded” in data that reflects participant experiences and perspectives [31]. Our reporting of this study’s methods and results is informed by the COREQ checklist [32].

### Setting

Fortaleza is the capital of Ceará state in northeastern Brazil and the fifth largest city in Brazil. It has an estimated 2021 population of 2.7 million people living in 119 neighborhoods (*bairros)* in six districts (*regionais*) [33]. Fortaleza has an equatorial savannah climate with a rainy season from February through April. Dengue has been endemic in Fortaleza for over thirty years, and all four serotypes have been found circulating in the city [34, 35]. Over the last decade, there were several years (2012, 2015, and 2016) in which more than 40,000 dengue cases in Fortaleza were reported [36, 37]. Cases dropped to roughly 10,000 annually in 2019, but rose dramatically in 2021 to more than 30,000 and reached nearly 40,000 in 2022 [37]. Far fewer cases were documented in 2020 (16,080), but this may be due to significant under-reporting during the COVID-19 pandemic. In epidemic years, dengue transmission follows seasonal patterns of increased intensity during the rainy season, while in non-epidemic years transmission persists beyond June [35]. Cases of Zika and chikungunya have been co-circulating in the city since 2015 [36].

### Data collection

We conducted in-depth interviews (IDIs) with Fortaleza residents who were sampled using a maximum variation strategy, a purposeful sampling strategy to identify individuals that vary on particular characteristics with the aim of representing diverse perspectives. Participants were purposively sampled across a range of education levels, and participants with and without a history of self-reported symptomatic infection with dengue in the past five years were sampled. Participants were recruited using several strategies. Students in health-related fields including medicine, nursing, and psychology at the Federal University of Ceará were recruited by a study investigator via messages sent to email listservs and Whatsapp groups. Hospital clinic patients were directly recruited by research assistants who also conducted interviews from three public hospitals: Walter Cantídio University Hospital, César Cals General Hospital, and Frotinha da Parangaba Hospital. Low-income community members were recruited by community health agents who presented the study and provided contact information for those who were interested in participating to the field research team. Snowball sampling was also used, with study participants providing contact information for friends, colleagues, or neighbors who might be interested in participating in the study.

Eligibility criteria included being a Fortaleza resident, at least 18 years of age, and providing informed consent to participate in the study. All interviews were conducted in a private location, and the average interview time was roughly 30 minutes (ranging from 20 to 45 minutes). The majority of interviews were conducted remotely, either on a telephone call (n = 21) or over a web-based video call platform (n = 9). The option of remote interviews was offered due to some participants’ concerns about the risk of COVID-19 infection during in-person interviews. Respondents were instructed to take these calls from a private location. Each of the in-person interviews (n = 13) was conducted in one of the three hospitals, except for one interview that was held in a preferred location in the community.

Interviews were conducted using a semi-structured interview guide with open-ended questions focused on knowledge of dengue, personal experiences with symptomatic dengue infection, risk perception of dengue, practices around dengue prevention, willingness to accept a hypothetical safe and effective dengue vaccine, and any anticipated changes in personal vector control behaviors after being vaccinated against dengue.

Interviews were conducted from April to June 2022 in Portuguese by a team of four research assistants (including the second author) overseen by the third author. All research assistants had approximately two years of experience conducting qualitative research under the supervision of the third author and are fluent Portuguese speakers. Two remote training sessions were conducted for the research assistants. The first training session covered research objectives, review of the consent form and interview guide, communication skills, and research ethics. Subsequently, research assistants conducted a total of ten pilot-testing interviews to refine the interview guide. The second training session included a discussion of the pilot-testing interviews and proposed updates to the interview guide.

All interviews were audio-recorded by the research assistant conducting the interview. Audio recordings were transcribed verbatim in Portuguese by a trained transcriptionist, and all transcripts were sent back to the interviewer who then compared the transcript to the audio recording for quality assurance. The second author translated all Portuguese transcripts verbatim into English. Interviews were continued until code saturation (the point at which no additional issues are being identified) was reached [38].

### Data analysis

Themes from the data were identified using an inductive approach, in which the data were iteratively compared and categorized. A team of two analysts (the first and second authors) independently coded and analyzed English transcripts using MAXQDA (2022.2.1) software. The first author developed the initial codebook based on reading all transcripts. The first and second author piloted and revised the codebook by conducting line-by-line coding on the same three transcripts. All remaining transcripts were divided between the first and second authors, who conducted line-by-line coding and wrote memos for each transcript. Data were triangulated across participants with and without symptomatic dengue infections in the past five years and across participants with varying levels of education. The first and second authors met weekly to make any revisions needed to the codebook, discuss emerging themes, and review any challenges throughout the coding and analysis process.

### Reflexivity

The data collection team was comprised of four Brazilian research assistants (three women and one man) led by a Brazilian study investigator who has a PhD and has lived and conducted research in Fortaleza for more than two decades. All research assistants were from Fortaleza and fluent in Portuguese. The second author completed all translations and collaborated with the first author on data analysis. The first author is a PhD student in global public health from the United States with previous experience leading malaria programs and research in East Africa and Southeast Asia and designing, implementing, and analyzing qualitative research. The first author did not travel to Brazil during data collection and did not have previous experience working in Brazil. To balance this lack of contextual knowledge, the first and second authors met weekly to discuss coding and analysis, with the second author providing insight and interpretation of the cultural context as a Fortaleza resident. The senior author is a Brazilian PhD-level demographer based in Boston whose research focuses on the identification of social, biological, and environmental risks associated with vector-borne diseases, particularly in Brazil. She provided additional insight into the epidemiology of dengue and the context of dengue control efforts in Fortaleza. All authors met monthly to review study progress and discuss interim findings.

### Ethical considerations

The study protocol was reviewed and approved by the Institutional Review Board of the Harvard T.H. Chan School of Public Health and the Ethics Committee of the Federal University of Ceará. Interviewers read informed consent forms in Portuguese. All participants were informed that their participation in this research was entirely voluntary and allowed to ask questions before being asked for verbal consent.

## Results

### Participant characteristics

We interviewed a total of 43 Fortaleza residents from a wide variety of educational and occupational backgrounds, ranging from 20 to 70 years old. Roughly three-quarters of our study participants self-reported a symptomatic dengue infection in the past five years. Study participant characteristics are presented in Table 1.

**Table 1:** Study participant characteristics

Characteristics		n (%)	
Study participants		43	
Sex			
Female		22 (51%)	
Male		21 (49%)	
Median age (years)		32	
Age groups (years)			
20–29	20 (47%)		
30–49	12 (28%)		
50–69	10 (23%)		
70+	1 (2%)		
Highest education level			
Incomplete basic education*	4 (9%)		
Completed secondary education	12 (28%)		
Current university student	17 (40%)		
Completed university	10 (23%)		
Marital status			
Single	23 (54%)		
Stable relationship	3 (7%)		
Married	13 (30%)		
Divorced	3 (7%)		
Widowed	1 (2%)		
Self-reported symptomatic dengue infection in the past 5 years			
Yes	33 (77%)		
No	10 (23%)		
Self-reported hospitalization for dengue infection in the past 5 years			
Yes	2 (5%)		
No	41 (95%)		

*Basic education refers to the first nine years of school in Brazil, inclusive of both primary and secondary years of education

### Knowledge of dengue

Knowledge of dengue symptoms, treatment, and transmission routes was strong among participants. It was widely known that dengue infections were transmitted by mosquitoes, with some respondents specifically naming the *Ae. aegypti* mosquito. While university students sometimes included more technical or clinical language in their answers (for example, referring to dengue as an *“arbovirus”* or the symptom of *“retro-orbital pain”*), participants across educational levels demonstrated generally good knowledge of dengue. Participants named myriad dengue symptoms including fever, chills, headache, rash, joint and muscle pain, and retro-orbital pain (pain behind the eyes). Accumulation of stagnant water, the presence of abandoned or vacant lots, and sanitation problems, such as poor garbage collection, were described in detail by respondents as causes of dengue transmission, with the understanding that these are conditions which facilitate the proliferation of mosquitoes.

### Personal vector control behaviors to prevent dengue

All participants described performing vector control activities in their own lives to prevent dengue. Fortaleza residents make significant efforts to prevent the accumulation of stagnant water in and around their homes, tipping over jars or bottles that might hold water, frequently changing the water in the saucers of potted plants, or avoiding houseplants altogether, and cleaning or paving over areas of their backyards or gardens that tend to hold standing water. Participants also reported that they are careful to dispose of garbage correctly and put it out on the street only on days when it is being collected.

“I think it’s really taking care of the water, getting water out of tires, cleaning and not letting water accumulate, keep changing water, try the water filter and everything else. Then the people in my case who had dengue again and who are in danger of having dengue hemorrhagic fever put those protective screens on the windows, be careful with the mosquitoes.” (Participant 20, male, university student)

Use of window screens, insect repellents, and long clothing was rarely mentioned; these were seen as additional measures that could be used to protect someone who had already been infected with dengue from a secondary infection.

### Perceived and desired government efforts to prevent dengue

Dengue prevention media campaigns on television, space-spraying (locally known as *fumacê*), and household visits by vector control agents to place larvicide in containers were described by participants as actions taken by various levels of government to prevent dengue. For each of these interventions, participants explained that they had witnessed these efforts in the past, but not recently—some people felt there was no government action currently being taken to prevent dengue. *Fumacê* was described as an ineffective but highly visible intervention, and a few participants noted that they were unsure about whether the government still conducted dengue prevention media campaigns because they don’t often watch television anymore, spending more time on social media.

Participants reported that government attention and resources had shifted away from dengue during the COVID-19 pandemic, and a previous period of renewed focus on *Aedes* mosquitoes with the emergence of Zika and chikungunya had been short-lived.

“Well, I think that with the pandemic of the coronavirus the campaigns against dengue ended up being a little left aside, you know both at the city level and the national level, even in the newspapers. So, I do not see much action from the government, so I think I cannot talk about what they are talking about because, at least, I do not see much action from the government.” (Participant 18, female, university student)

There was a strong desire among participants for greater government-led action to prevent dengue, particularly at the municipal level. Participants raised the need for renewed media and education campaigns, improved infrastructure such as street cleaning services, and better surveillance and control programs for common *Ae. aegypti* breeding grounds such as vacant lots and stagnant bodies of water. Dengue was seen as a particular problem in the city’s periphery, with some participants emphasizing the need for government intervention to improve sanitation infrastructure:

“Dengue is more prevalent in the periphery, in needy neighborhoods, in sewers and where there is open sewage…As long as there is no basic sanitation, as long as there is no adequate infrastructure to be able to fight the health situation itself, there is no fight against dengue.” (Participant 40, male, self-employed)

### Dengue vaccine acceptability and motivating factors

Interviewers asked study participants to consider the following scenario: *“A new safe and effective vaccine offers protection against dengue. It becomes available for free. Would you take this dengue vaccine? Why or why not?”* Interviewers went on to ask whether and why or why not participants would vaccinate their children—or, if they had no children, future children or young family members—with this dengue vaccine.

Overall acceptance of a hypothetical dengue vaccine was strong, with a high willingness among participants to receive the vaccine. Participant responses to the question of whether or not they would vaccinate children against dengue generally mirrored their responses to the question of whether or not they would vaccinate themselves. Participants emphasized that their vaccine acceptance was dependent on the vaccine’s safety and effectiveness. While participants generally did not define what they would consider to be a safe and effective vaccine, some expressed a desire for a high degree of certainty on the vaccine’s safety and effectiveness. For example, one woman said that she would be willing to take a dengue vaccine, “*If I was sure, certain that it is effective and safe I would take it.” (Participant 43, female, cleaning assistant)*. Some participants asserted they wanted a vaccine with no side effects, while others acknowledged that vaccines are likely to have mild side effects. More generally, participants described that they wanted to know that the vaccine had been appropriately tested and approved.

We identified four broad motivating factors behind dengue vaccine acceptance: perceived susceptibility to dengue infection, negative personal experiences with dengue illness, a desire to protect oneself and others against dengue, and reported trust in scientific and regulatory processes to ensure vaccine safety and effectiveness.

#### Perceived susceptibility to dengue infection

Participants reported that dengue was a significant health problem in their neighborhood and perceived themselves and their communities to be at high risk of infection.

“Look, I think we all are at risk - I’ll tell you why. I have never thought or imagined that I was going to get dengue, because where I lived, in the neighborhood that I used to live in the condominium, the staff is highly rigid about this question of cleaning and stuff like that and yet I got it…But, of course, everyone is liable to get it, from even those who live in the Aldeota to those who live in the most vulnerable neighborhood, why? Because all it takes is a little bit of stagnant water or something like that for the mosquito to thrive, right?” (Participant 14, male, retired)

This sense of widespread susceptibility to dengue infection influenced participants’ willingness to receive a dengue vaccine. This is exemplified by the perspectives of the only two study participants who indicated that they would not be willing to immediately receive a dengue vaccine. One of these vaccine-hesitant participants explained that she did not feel at sufficient risk of dengue infection to get vaccinated (and was already taking other precautions), while the other would delay vaccination due to concern that the risk of side effects from a new dengue vaccine would be higher than the risk of dengue infection itself.

“At the moment there is no risk, thank God everything is fine…I’m not afraid of catching it because we are very careful, right?” (Participant 39, female, maid)

“Because I’m very afraid of like using new things. I would let several people go first, if they didn’t feel anything or if it was really effective then I would get it…because there are things that I am more afraid of, the reactions that I would possibly have than the disease itself, maybe the reactions are even worse than dengue, you know? So, I think I would let a lot of people get it, and then I’d take it.” (Participant 21, female, realtor)

The former study participant, who had not experienced a symptomatic dengue infection and did not feel at risk, clarified that she would be willing to receive a dengue vaccine *“if there was a real danger, if there was a real risk of catching the disease, you know?”* The latter participant had experienced a symptomatic dengue infection, but still weighed the potential risks of a new vaccine as being higher than the risk of dengue infection.

#### Negative personal experiences with dengue illness

Participants who had experienced a symptomatic dengue infection in the past five years described painful and negative experiences with the disease that influenced their willingness to be vaccinated against dengue. Strong physical pain and discomfort, with symptoms including fever, chills, headache, myalgia, retro-orbital pain, and rash, were often accompanied by substantial worry and fear about the risk of hemorrhage or death. While only two study participants had been hospitalized for their dengue infection, some of those who had been sick with dengue noted that they had very low platelet levels or that they were surprised or alarmed by the severity of their symptoms. Other concerns such as chronic health conditions, allergies to common painkiller medications, and inconvenience and stress caused by time away from work affected participants throughout the course of their illness with dengue and motivated them to accept a dengue vaccine.

Concern about the higher risk of severe illness and death with secondary dengue infections was high among participants who self-reported a dengue infection in the past five years. Participants who had experienced dengue symptoms described dengue as a potentially deadly disease in explaining their willingness to be vaccinated.

“I, who have had dengue fever, I know how painful and dangerous dengue is…mine I got cured at home, but I know that people die every year of dengue. I have people in my family who have had hemorrhagic dengue who almost died and I do not want to go through that scare, I do not want to face every year the fear of catching it. If there was a vaccine that protected me, I would want to.” (Participant 27, female, notary)

While people without personal experiences of symptomatic dengue illness were generally willing to be vaccinated, they did not convey the same intensity of concern about the risk of severe illness and death as those who had experienced symptoms.

#### Desire to protect oneself and others against dengue

Study participants asserted in definitive language that they would want to receive this hypothetical dengue vaccine using terms like: *“for sure,” “absolutely,” “definitely,” “no problem,”* and *“of course.”* For some, it was an obvious choice to accept a dengue vaccine that would protect themselves and others from dengue infection, or at least from severe outcomes. Dengue vaccination was seen as one important way to contribute to disease control efforts, with beliefs on dengue vaccination often described in terms of personal experiences of the COVID-19 pandemic.

“Because as it was with COVID, if we have a wall to protect ourselves…if we have a way to avoid dengue that in some cases can be serious, we do it, we use these means for prevention.” (Participant 19, male, university student)

Dengue vaccination offered a way to take individual action against an infectious disease risk that is heavily mediated by the surrounding environment and the actions of others—government officials, neighbors, and family members—outside of study participants’ control. Across the interviews, a sense of powerlessness emerged concerning dengue exposure. As residents of a dengue-endemic city also affected by Zika and chikungunya, some participants felt that they are always vulnerable to vector-borne infection, no matter what they do. Dengue vaccines would offer a new means for individuals to take control over their own disease risk. One social worker described this sense of helplessness and her efforts to prevent dengue:

“…I started to use repellent all the time, you know, in the house, me and my son, but we had already been bitten by the mosquito. But I’m stuck with this feeling of powerlessness. I think the mosquito arrives differently from Covid that we put on the mask, that we are with distance, that we take care of ourselves, but [with] this mosquito, I had a feeling of helplessness and there was nothing I could do besides the care of not accumulating water inside the house, so I feel like that.” (Participant 32, female, social worker)

She went on to explain that she would take a dengue vaccine, vaccinate her children, and raise vaccine awareness among others.

#### Trust in scientific and regulatory processes

As participants explained why they were willing to take a dengue vaccine, they referenced their belief or sense of trust in science broadly and vaccines specifically, occasionally citing Brazil’s National Health Surveillance Agency (ANVISA) by name. Participants invoked other vaccination campaigns, explaining that they would accept a dengue vaccine in the same way that they would accept other routine vaccinations. These beliefs had sometimes been passed down through families since childhood. Again, personal experiences with the COVID-19 pandemic permeated discussions on dengue vaccination. Receiving a COVID-19 vaccine was described as both a political act and an act of faith, which some participants then applied to the question of dengue vaccination.

“I think that the vaccine for me is much more personal. We are part of a group, right, and when I went to get the vaccine for Covid I said aside from being an individual collective issue at that moment it was also a political act…Because vaccines were such a common thing, and I think people didn’t even care to take or not. I never noticed vaccines like this, before this whole debate of science denialism regarding Covid, I never saw a controversy like that, never. So, I think that getting the vaccine is in fact an individual action and a collective action, because the more, imagine the population of Brazil everyone being vaccinated against dengue, then the mosquito wouldn’t have many people to be transmitting dengue here. And certainly, it is of utmost importance for me, any vaccine.” (Participant 32, female, social worker)

None of the participants mentioned the existing Dengvaxia vaccine or the development of new dengue vaccines.

### Anticipated impact of dengue vaccination on personal vector control behaviors

Study participants were asked whether they expected either the type or the frequency of their current dengue prevention activities to change in any way if they were vaccinated against dengue. Study participants varied in their perspectives on the imagined impact of dengue vaccination on their own vector control behaviors. Among those who reported that they would not expect anything about their current vector control activities to change after being vaccinated against dengue, participants explained that they believed that no vaccine would provide complete protection, either against infection or against all dengue serotypes. Some study participants saw household vector control measures as an essential part of household cleanliness and hygiene that would be necessary regardless of the risk of dengue exposure, and the ongoing risk of chikungunya and Zika was also considered. When one woman was asked whether she would change anything about her personal vector control efforts after dengue vaccination, she responded:

“No, not me, because it is a habit of mine, of my family, both my husband’s side and mine and my family’s side, we have always been very careful with the issue of hygiene and not only because of dengue, but also because of other diseases that this lack of care can bring.” (Participant 24, female, self-employed)

Other participants felt that they would gain a sense of protection from a dengue vaccine that might change their personal vector control behaviors, although only one participant expected to eventually quit all vector control efforts after being vaccinated and not immediately. Similarly to the discussions around acceptance of dengue vaccination, study participants used their experiences with COVID-19 vaccination to imagine how they might feel about continuing their vector control behaviors after being vaccinated.

“…using my intelligence I think that even vaccinated you should still continue with the culture of preventing the proliferation of mosquitoes, right? But, as a human being I know that the human being begins over time to loosen up, neglect, as it is in the pandemic too, everyone got the vaccine and everyone is wearing a mask and everything and even today I wear a mask, but I know that over time I will no longer wear a mask.” (Participant 26, male, self-employed)

## Discussion

This study gathered qualitative data on the beliefs and perspectives of Fortaleza residents with a range of educational backgrounds and personal experiences with dengue illness to examine dengue vaccine acceptability and the anticipated effects of dengue vaccination on personal vector control behaviors. Participants demonstrated strong knowledge that dengue is a mosquito-borne illness and were able to name common symptoms of dengue. Dengue was seen as an important health risk in their communities, and participants described significant efforts to control mosquitoes in or near their homes, particularly by preventing the accumulation of stagnant water. Dengue vaccine acceptance was high among our study participants; however, vaccine safety and effectiveness were emphasized as pre-conditions to this acceptance. Perspectives on the anticipated impact of receiving a dengue vaccine on personal vector control behavior varied—while some study participants saw household vector control as an essential part of cleanliness and protection against other arboviruses, others expected to gain a sense of protection from dengue vaccination that might influence how they attempt to control mosquitoes in or near the household.

Our study contributes to a limited body of research available on dengue knowledge, attitudes, and practices in Brazil conducted in the past two decades [39–41]. It also extends an existing body of literature on dengue vaccine acceptance primarily conducted in Asia to a large urban center in Brazil. Only a small number of studies have been conducted on dengue vaccine acceptance [26, 27] and willingness-to-pay for dengue vaccines in Indonesia [26, 28], the Philippines [30], and Vietnam, Thailand, and Cambodia [29]. Vaccine hesitancy is often driven by concerns about safety and possible side effects, as seen in hesitancy surrounding HPV vaccines [42–44] and, more recently, COVID-19 vaccines [45, 46]. While none of our study participants mentioned the Dengvaxia vaccine, it is difficult to predict what vaccine hesitancy issues gain traction and concerns about one vaccine can deepen hesitancy towards other vaccines, as seen in declining measles vaccination in the Philippines after outrage erupted in response to the Dengvaxia safety issues [19].

This study identified four motivating factors for dengue vaccine acceptance: perceived susceptibility to dengue infection, negative personal experiences with dengue illness, a desire to protect oneself and others against dengue, and reported trust in scientific and regulatory processes to ensure vaccine safety and effectiveness. Our analysis relied on inductive coding to ensure that results would be grounded in study participants’ own experiences and perspectives. However, the themes emerging from this data strongly overlapped with components of the Health Belief Model, a theoretical framework that has been widely used to explain health behaviors, including vaccination. According to the Health Belief Model, four key health beliefs—perceived susceptibility to illness, perceived severity of illness, perceived benefits of behavior change, and perceived barriers to behavior change—directly relate to health behavior [47, 48]. Later, two additional constructs were added to the model: cues to action (a stimulus to trigger decision-making to accept a health action) and self-efficacy (a person’s level of confidence in their own ability to successfully perform a behavior) [48].

The pervasive sense of risk of dengue infection described by our respondents was a strong motivator to vaccinate against dengue and control mosquitoes at home, in alignment with the Health Belief Model’s first component of perceived susceptibility to illness. Similarly, reported negative personal experiences with dengue illness map onto the Health Belief Model’s second component of perceived severity of infection. Participants who had experienced symptomatic dengue vividly described painful symptoms and resulting fear of illness and death that influenced their perception of dengue as a common but severe illness warranting measures of protection. Study participants also described perceived benefits of behavior change (in this case, accepting a hypothetical dengue vaccine), the third component of the Health Belief Model. Much of their reported willingness to accept a dengue vaccine was motivated by the perceived benefits of protecting themselves and their family members from dengue illness and, more broadly, protecting their communities from dengue epidemics. Discussion on perceived barriers to vaccination—the fourth component of the Health Belief Model—was more limited; however, the hypothetical dengue vaccine we proposed for consideration was described as free, safe, and effective, eliminating some potential barriers such as cost. The importance of generating and clearly communicating rigorous evidence around vaccine safety is already well understood, but the emphasis that our study participants placed on vaccine safety and effectiveness serves as a reminder that Fortaleza residents are sensitized to these issues and gaps in evidence or knowledge, as well as misinformation, could present significant threats to dengue vaccine uptake.

Reflecting on their own current efforts to prevent dengue—through household vector control and without a vaccine—some participants expressed a sense of hopelessness which relates to the Health Belief Model’s concept of self-efficacy. While participants invested significant time and resources into controlling mosquitoes at home, some felt that it was difficult or nearly impossible to protect themselves against a vector-borne disease endemic throughout the city and thriving in areas with poor infrastructure for waste and sanitation. For these participants, dengue vaccines may offer an important opportunity to take control of the persistent threat posed by dengue.

Study participants reported familiarity with several government efforts to prevent dengue transmission, such as media campaigns on television and household visits by community health agents. However, there was frustration with perceived gaps in these interventions in recent years, mirroring earlier qualitative research in Brazil which found that community members perceive dengue prevention and control efforts by municipal authorities to be inadequate [49]. Brazil’s Ministry of Health reported in December 2020 that vector control measures including insecticide distribution, home visits by health agents, and community education programs about stagnant water had been delayed due to COVID-19 [50]. Reflecting these disruptions, study participants felt that dengue had been forgotten by government and public health authorities in the wake of the COVID-19 pandemic.

Personal experiences with COVID-19 are woven throughout the interviews in this study, shaping how study participants understand and explain their feelings about dengue vaccination and how they imagine being vaccinated against dengue might influence their vector control behaviors. While many of the emerging themes in this study are well described by the Health Belief Model, our study participants’ continual reflections on their experiences of this global crisis are not captured by any one element of the Health Belief Model. Experiences of the COVID-19 pandemic may continue to inform individuals’ vaccine and vector control decisions for other infectious diseases such as dengue. Study findings also demonstrate the importance of social norms in vaccine decision-making, an element that is not included in the Health Belief Model, with study participants relating a general sense of trust in science and Brazilian regulatory authorities to dengue vaccine acceptance.

Perspectives on the potential impact of dengue vaccination on vector control behaviors were varied in this study. Study participants who felt that there was an ongoing risk of chikungunya and Zika and that vector control measures are part of good household cleanliness did not expect to change their vector control behaviors after receiving a dengue vaccination, but others anticipated feeling a sense of protection from dengue vaccination that might change how often they take measures to reduce accumulated water near the household. Results from a community-based survey in Indonesia reporting high levels of acceptance for a hypothetical safe and effective dengue vaccine (94.2%) suggested a small potential impact of dengue vaccination on attitudes towards vector control, with 7.2% of respondents stating that vector control would not be necessary if a dengue vaccination program was available [26]. Perceived susceptibility and severity of these other arboviruses may play a key role in influencing household vector control measures after the introduction of a dengue vaccine. Until a dengue vaccine is scaled up across Brazil, questions around household vector control following dengue vaccination are hypothetical ones that warrant future research.

This study has some limitations. First, these qualitative results provide insight and perspectives from diverse respondents, but cannot be broadly generalized to all Fortaleza residents or residents of other parts of Brazil. Only two respondents in this study sample expressed hesitancy around receiving a hypothetical safe and effective dengue vaccine, limiting our ability to draw meaningful conclusions about the specific underlying reasons for potential dengue vaccine hesitancy. Second, participants were presented with a hypothetical vaccine described as “safe” and “effective” in open-ended terms, without quantification. Study participants may have differed in their interpretation of what would qualify a vaccine as “safe” and “effective.” Third, self-reported willingness to receive a dengue vaccine and continue vector control behaviors may not correlate well with future vaccine and vector control decisions, which are complex and may change in unpredictable ways over time. Reports of expected vaccination and vector control behaviors may also be influenced by social desirability bias.

## Conclusions

Among the Fortaleza residents interviewed for this study, there was a high willingness to accept dengue vaccination, given that the vaccine would be free and demonstrably safe and effective. Respondents expressed diverse perspectives on how receiving a safe and effective dengue vaccine might influence their personal vector control behaviors and related these predictions to their perception of risk from other *Aedes* mosquito*-*carried infections and beliefs about the relationship between vector control and household cleanliness. With the introduction of any novel dengue vaccine in Brazil, it will be essential to clearly communicate available evidence on vaccine effectiveness and safety, including expected side effects. Future research should explore dengue vaccine uptake and drivers of vaccine acceptancy and hesitancy in the real-world context of a novel vaccine introduction, and healthcare providers should be equipped with locally relevant data to respond to possible vaccine concerns with accurate information. Potential changes in vector control behavior at the individual and household level with the introduction of a dengue vaccine should also be monitored. Renewed efforts are needed to encourage community members to continue efforts to control *Ae. aegypti* mosquitoes to optimize the impact of vaccinations and prevent rising cases of other arboviruses such as Zika and chikungunya. At the same time, there is a pressing need for infrastructure improvements to ensure routine access to piped water, garbage collection, and sewage systems. Without urban infrastructure improvements, Fortaleza residents will continue to be threatened by arboviruses carried by *Aedes* mosquitoes, even if they are vaccinated against dengue and proactive in their own vector control efforts.

## Data Availability

The datasets used and/or analysed during the current study are available from the corresponding author upon reasonable request.

## Abbreviations

DENV: Dengue virus
IDI: In-depth interview
WHO: World Health Organization
